# BidSi6 and BidEL isoforms as a potential marker for predicting colorectal adenomatous polyps

**DOI:** 10.1186/s12920-022-01282-0

**Published:** 2022-06-06

**Authors:** Flora Forouzesh, Fatemeh Sadat Kia, Ehsan Nazemalhosseini-Mojarad

**Affiliations:** 1 Department of Genetics, Faculty of Advanced Science and Technology, Tehran Medical Sciences, Islamic Azad University, P.O. Box: 193951495, Tehran, Iran; 2 Department of Cancer, Gastroenterology and Liver Disease Research Center, Research Institute for Gastroenterology and Liver Diseases, Shahid Beheshti University of Medical Sciences, Tehran, Iran

**Keywords:** Adenomatous polyposis, Bid, Isoforms, Prognosis, Colorectal cancer

## Abstract

**Background:**

As a well-known protein, Bid links the extrinsic and intrinsic apoptotic pathways and plays important roles in cell proliferation. In this study, we evaluated the expression of two isoforms of the Bid gene (BidSi6 and BidEL) in colorectal adenomatous polyps as a biomarker and investigated the relationship between their expression levels with clinicopathological factors.

**Methods:**

The expression of BidSi6 and BidEL isoforms in 22 pairs of Adenomatous polyps and adjust non-polyp tissues was measured by qReal-Time PCR and compared with 10 normal colon tissues. ROC curve was performed to examine the diagnostic capacity. Also, sequencing was performed for molecular identification of BidSi6 isoform in adenomatous polyp.

**Results:**

Our results showed that BidSi6 and BidEL isoforms were significantly overexpressed in Adenomatous polyps and non-polyp adjacent tissues from the same patients compared to that in normal colon tissues, but there was no significant expression between polyps and adjust non-polyp tissues. There were no significant correlations between the expression of two isoforms and other features of clinicopathology. The area under the curve of BidSi6 and BidEL isoforms indicated powerful diagnostic capability. The phylogenetic tree was constructed based on the sequence of idSi6 isoform, and the results showed that adenomatous polyp tissue and adjust non-polyp tissue were separated from healthy colorectal tissue and reference sequence (EU678292).

**Conclusions:**

These findings suggest that BidSi6 and BidEL isoforms can be used as new potential biomarkers in adenomatous polyps.

**Supplementary Information:**

The online version contains supplementary material available at 10.1186/s12920-022-01282-0.

## Background

Colorectal cancer (CRC) occurs mainly in non-cancerous adenomas or polyps that form on the colon and rectum. Epithelial polyps are classified as adenomatous or hyperplastic (HP), and adenomatous polyps are considered progressive lesions [1]. Evidence indicates that alternative splicing plays a crucial role in cancer. More than 1500 splice events in 885 genes were reported and most of them enriched CRC-associated pathways and functions [2]. These events affect the carcinogenesis of colon cancer [3]. Alternative splicing may be used as biological markers of prognosis and diagnosis [4].

The Bcl-2 family greatly influences the progression of cancer and regulators of cell death, hence affecting the sensitivity of tumor cells to radiotherapy.

Bid is a member of Bcl-2 family protein and impacts several pathways of apoptosis by incorporating signals in mitochondrial apoptosis [5, 6]. Growing evidence supports that Bid has critical roles for stress-response [7, 8]. Alternative splicing in the Bid gene might affect the function of the gene. The isoforms have differences in expression, functional effects and cellular localization. There are three Bid splice variants and tissue distribution of three proteins is exclusive and distinct. Most importantly, they modulate cellular apoptosis differently [9]. BidS isoform has the N-terminal inhibitory fragment of Bid and does not have the BH3 domain. BidL (full-length) which denominated BidEL has an additional N-terminal sequence. BidES isoform has only the downstream of the BH3 domain of the Bid sequence. BidS functions as an inhibitor of apoptosis, whereas BidEL induces apoptosis. BidES induces apoptosis but is also able to partially inhibit the pro-apoptotic function of truncated Bid [6].

Previous studies indicate that the balance between gene variants seems to regulate cellular proliferation, differentiation and apoptosis, and the inhibition of tumor growth. Obtaining splicing patterns in malignancies will provide novel prognostic and diagnostic biomarkers and introduce novel treatments in cancer therapeutic targets. In other words, the splice variants that are involved in tumor formation and development can be targeted by molecular therapies that are close to clinical usage These therapies include modifying the splicing events by the use of oligonucleotide-mediated therapies, targeted protein therapy by small peptides of protein isoforms or splicing regulators, and reprogramming of RNAs to modify alternative splicing patterns [10–12].

The conventional therapies of CRC are surgery, chemotherapy, and radiotherapy. Also, the combination of chemotherapy and immunotherapy targets the dysregulated proteins [13]. Colonoscopy is a main diagnostic tool to screen for CRC since it can detect and effectively remove pro-malignant and malignant lesions. Approximately all gastroenterology and cancer societies recommended this test, following a positive fecal occult blood test, to be performed every 10 years in people of average risk starting from the age of 50 [14]. To help clinicians to optimize their function, it is important to nominate more effective tools that improve early diagnosis and predict the most likely progress of the disease and response to chemotherapy. Thus, they mitigate both the morbidity and mortality of their patients. So, researchers are now developing novel therapeutic approaches for the treatment of CRC by detecting important key biomarkers to understand their molecular mechanisms [15].

In this study, we investigate the expression of two isoforms of Bid (BidEL and Bidsi6) transcript in adenomatous polyps and adjust non-polyp tissues from the same patient compared to healthy controls. The study objective is also to assess the expressions of isoforms of Bid in adenoma depending on the sex and age of patients, tumor location in the colon and rectum, and the effect of BidEL and BidSi6 expression in adenomatous polyps.

## Materials and methods

### Patients

The sample was chosen from the cases with adenomatous polyps that were referred to the Taleghani Hospital, Tehran, Iran, from April 2014 to May 2016. We collected 22 adenomatous polyps and adjacent non-polyps tissues and 10 healthy colorectal tissue as controls. Biopsy samples of the adenoma polyp of patients were confirmed by a pathologist. The patients had not received any chemotherapy before the surgery. The patients’ age ranged from 28 to 75 years with a mean ± SE of 52. The healthy controls were selected from biopsy samples with a disease-free health as confirmed by a pathologist. The patients that participated in this study provided signed informed consent by the Research and Ethical Committee of Taleghani Hospital. The information about the clinicopathological characteristics of all contributors was retaken from medical records and questionnaires (Table 1).

**Table 1:** Demographic and clinical characteristics of the cases

Characteristics	Variable	Frequency, n (%)
Sex	Male	14 (63.6%)
	Female	8 (36.6%)
Age	< 50	9 (40.9%)
	> 50	13 (59.09%)
Family history	Yes	8 (36.6%)
	No	14 (63.6%)
Polyp’s location	Ascending colon	4 (18.1%)
	Transverse colon	6 (27.2%)
	Descending colon	5 (22.75)
	Sigmoid	1 (4.5%)
	Rectum	6 (27.2%)

### Isolation of total RNA

Total-RNA from tissues was extracted using the Yekta Tajhiz Azma kit (Iran, Tehran) according to the manufacturer's instructions. Quantification of total RNA was determined through spectrophotometry (Thermo Nanodrap).

### Reverse transcription polymerase chain reaction

cDNA synthesis was performed with TaKaRa kit (Cat No.RR037A, Tokyo, Japan) according to the manufacturer's instructions. For this purpose, 2000 ng of total RNA was denatured at 95 °C for 5 min. Then, tubes were placed on ice and 5 μL of 5 × primer script buffer, 0.5 μL RT enzyme (AMV Reverse Transcriptase XL; 5 U μl^−1^), 1.24 μM oligo dt primer, 10 μM random6 mer, 1 μM ribolock, 1 μL easy dilution, and 5 μL RNA free distilled water (dH2O) were added. The final reaction volume was 20 μl. The reaction mixture was incubated at 25 °C for 5 min, 42 °C for 15 min, and 85 °C for 1 min; then, the prepared cDNA was stored at − 70 °C until use.

### Real-Time quantitative polymerase chain reaction

Quantitative Real-Time PCR was performed using the SYBR Premix Ex Taq II kit (TaKaRa Biotechnology, Japon) in a 7500 Real-Time PCR System (Applied Biosystems, CA, USA). Two pairs of gene-specific primers (BidSi6 and BidEL) were designed using the Gene-runner version 3.05 software. The BidSi6 real-time PCR primers were 5′- AATAGAGGCAGGGGCGTC -3′ and 5′- TCAGTCCTCCTCCTCTGGC-3′, producing a 157-bp PCR amplicon. The BidEL real-time PCR primers were 5′- AAGTGGCTGGGCTGGCAAG -3′ and 5′- CCAGTGGCGACAGAATCCG -3′, producing a 138-bp PCR amplicon (Additional file 1). The Beta-2-microglobulin real-time PCR primers were 5′- TGCTGTCTCCATGTTTGATGTATCT -3′ and 5′- TCTCTGCTCCCCACCTCTAAG -3′, producing a 86-bp PCR amplicon.

The reaction mixture contained 1 µl of each primer (10 pmol), 5 µl of cDNA, 10 µl of SYBR Premix Ex Taq II (2x), 0.3 µl of ROX Reference Dye (50x) and 3.7 µl of sterile distilled water, in a final reaction volume of 20 μl. The cycling conditions were as follows: 95 °C for 30 s, followed by 40 cycles of 95 °C for 5 s and 60 °C for 34 s. Beta-2-microglobulin was used as an internal control gene to normalize the PCRs for the amount of RNA added to the reverse transcription reactions. The relative expression levels of BidSi6 mRNA and BidEL mRNA were calculated by the 2^–∆∆Ct method. Each real-time PCR reaction was performed in triplicate to evaluate the reproducibility of data.

### Sequencing of BidSi6 isoform

#### Conventional PCR

For sequencing, other reverse BidSi6 primers were designed using the Gene runner (version 3.05) software at the junction of the exon to exon. The following factors were considered in the design of the primer: (1) primer-dimerization does not form. (2) BidSi6 primers must amplify a unique region as the primers itself is unique. (3) BidSi6 primers in the same direction must be non-overlapping. (4) The primer selection must be physicochemically appropriate, as described by GC content, melting temperature and other parameters. The sequence of Forward BidSi6 primers is 5′- AATAGAGGCAGGGGCGTC -3′ and Reverse BidSi6 primers is 5′- 5TGCTACGGTCCATGCTGTC-3′. The size of PCR fragment is 777 base pair.

For subsequent PCR analysis, 1 μl of the first-strand cDNA was used for PCR amplification in a reaction volume of 25 μl. All PCR reactions PCR were performed using the Sinaclone kit (Tehran, Iran). The following PCR conditions for BidSi6 gene were used: initial denaturation performed at 95 °C for 5 min, followed by 40 cycles where denaturation occurred at 94 °C for 45 s with an annealing temperature of 62 °C for 40 s. Elongation was performed at 72 °C for 30 s and the final extension at 72 °C for 5 min. Negative control with primers was used for all the PCR amplifications. Then, the PCR products were separated in 1.5% agarose gel electrophoresis and visualized under UV light (Vilber Iourmat, UK).

#### Nucleotide sequencing

The positive results in electrophoresis were selected for sequencing. Distinct gel bands were purified with a gel extraction kit (Qiagen- Germany). Analysis of sequences was performed using the ChromasPro (version 2.6.5) software. All the nucleotide sequences were aligned with each other. According to the identity percentage and query coverage parameter, the reference sequences were downloaded from the GenBank database using the Basic Local Alignment Search Tool (BLAST), Claustal omega (https://www.ebi.ac.uk/Tools/msa/clustalo/), and other programs available in NCBI site (National Center for Biotechnology Information) to determine the isoforms of BidSi6.

#### Nucleotide sequence accession numbers

If the isoform BidSi6 obtained in this study with the cutoff values above 95% in nucleotide and amino acid sequence similarity was identical to those published in GenBank, these sequences were identified to be known isoforms and would be registered in the GenBank and given the first published name.

### Statistical analysis

All the results were recorded as means ± standard deviation and the statistical analyses were conducted using the GraphPad Prism version 3.05 Software (GraphPad Software, CA, USA). Student's t-test and one-way analysis of variance (ANOVA) were used. To assess the diagnostic performance of BidSi6 and BidEL, the Receiver operating characteristic (ROC) curve and the area under the ROC curve (AUC) were constructed. *P* < 0.05 was considered statistically significant.

## Results

### Bidsi6 mRNA expression in adenoma polyp tissues

**Fig. 1:**
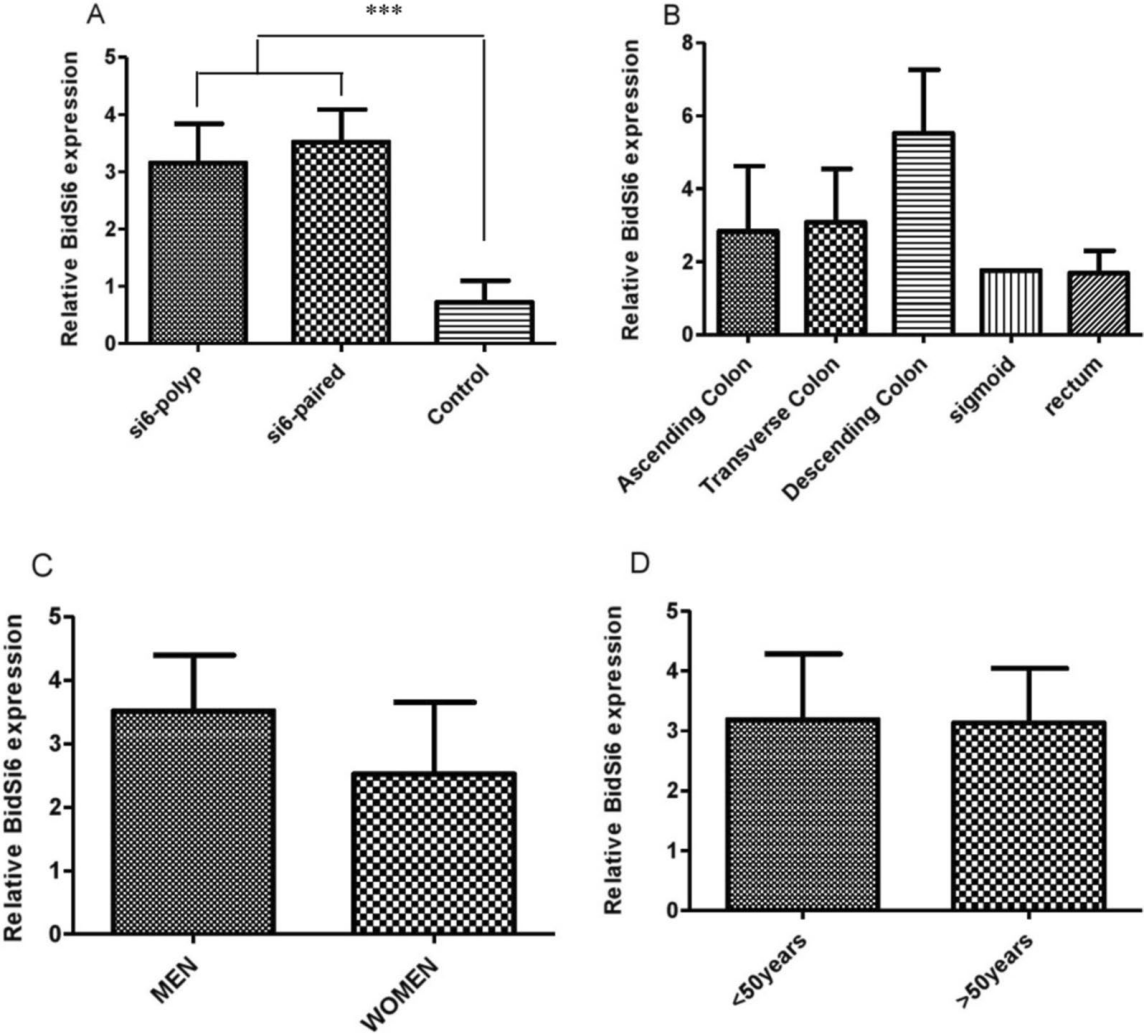
The expression level of BidSi6 isoform. **A** The expression of BidSi6 mRNA in adenomatous polyps and adjust non-polyp tissues (paired) and normal samples (control). **B** Comparison of BidSi6 mRNA expression in polyps located in colon and rectum and normal samples. **C** Comparison of BidSi6 gene expression between two sexes. **D** The expression of BidSi6 in patients with higher than 50 years and less in age. Each value is the means ± S.E. of three separate experiments. ***P < 0.001, significantly different from normal samples

BidSi6 isoform expressions were measured in 22 pairs of adenomatous polyp tissues and adjust non-polyp tissues from the same patient, and the expression values obtained were compared between the paired samples and healthy colorectal tissues. We found that BidSi6 isoform expression increased significantly in both adenomatous polyp tissues and the paired adjust non-polyp tissues compared with the control group (healthy colorectal tissues) (*P* = 0.0007) (Fig. 1A). BidSi6 was down-regulated in adenomatous polyp tissue samples compared to adjust non-polyp, but the difference in expression between them was not statistically significant (*P* = 0.668).

### Correlation of BidSi6 expression with clinicopathological features of patients with adenomatous polyp

We compared the BidSi6 expression with clinicopathological features of adenomatous polyp between these groups. According to the location of the adenomatous polyp, the study group was divided into five subgroups: patients with polyps located in ascending colon, transverse colon, descending colon, sigmoid and rectum. Polyps were seen in ascending colon, transverse colon, descending colon and rectum showed increased expression compared to the control group. However, in the sigmoid section of the intestine and rectum, there was no change in expression of the *BidSi6* gene compared to the control group (healthy colorectal tissue) (Fig. 1B). We found that the expression of BidSi6 in descending colon location was higher than other polyp’s location.

We compared the mRNA levels of BidSi6 between male and female patients. The expression of the Bidsi6 isoform in the male group was higher than in the female group, but there was no significant difference between them (*P* = 0.497) (Fig. 1C)*.*

To better understand the relationship between BidSi6 expression and patient age, patients were divided into two groups (≤ 50 years and > 50 years). No significant difference was observed between ≤ 50-year and > 50-year groups (*P* = 0.969) (Fig. 1D).

### BidEL mRNA expression in adenomatous polyp tissues

**Fig. 2:**
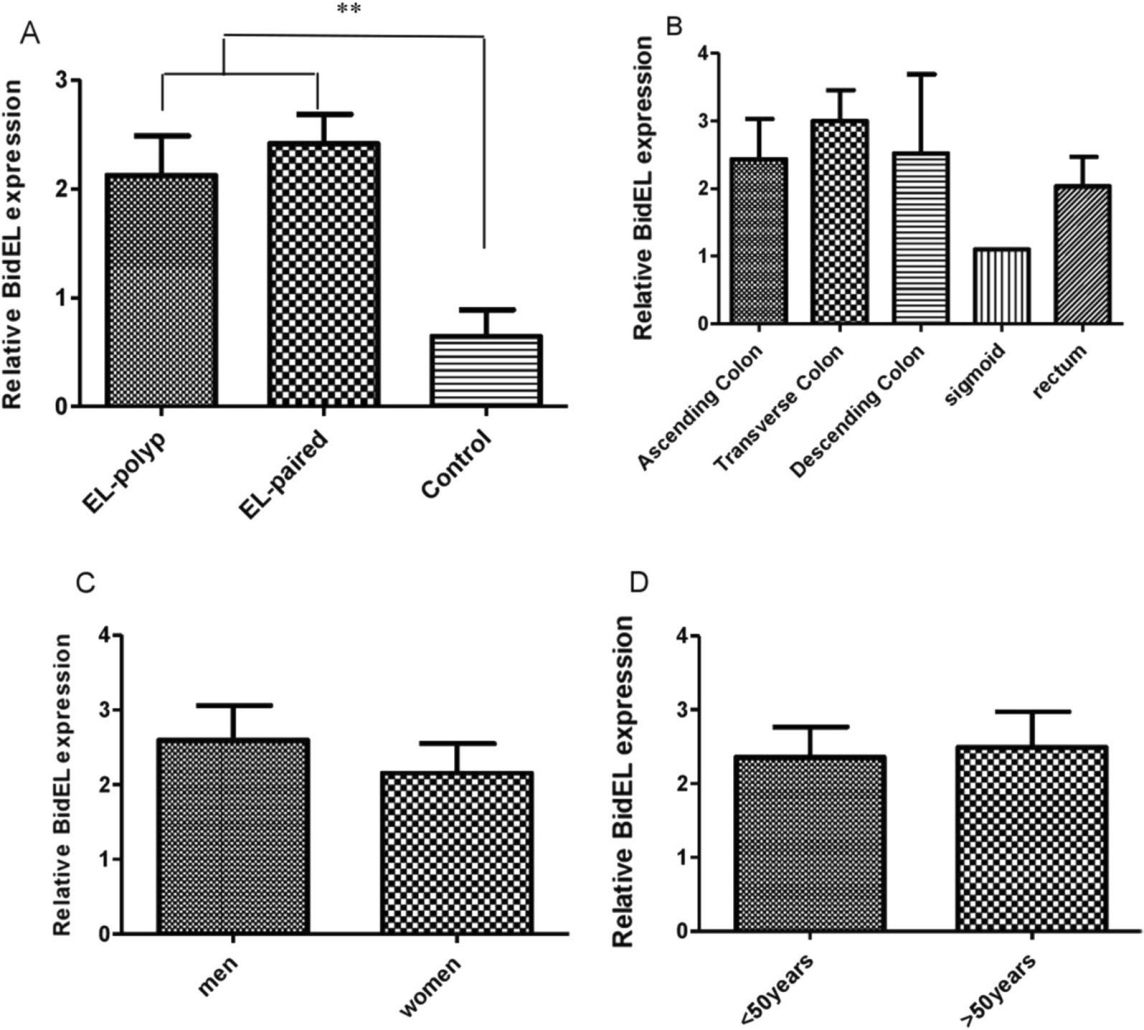
The expression level of BidEL isoform. **A** The expression of BidEL mRNA in adenomatous polyps and adjust non-polyp tissues (paired) and normal samples (control). **B** The expression of BidEL in patients higher than 50 years and less in age. **C** Comparison of BidEL gene expression between two sexes. **D** Comparison of BidEL mRNA expression in polyps located in colon and rectum and normal samples. Each value is the means ± S.E. of three separate experiments. (**P < 0.01), significantly different from normal samples

Expression of BidEL was significantly higher in adenomatous polyp tissues and adjacent non-polyp tissues than in control groups (healthy colorectal tissues) (Fig. 2A) (*P* = 0.035). Also, BidEL was down-regulated in adenomatous polyp tissue samples in comparidon with adjacent non-polyp tissues, but the difference in expression between adenomatous polyp tissue samples and adjust non-polyp tissues samples was not statistically significant.

### Correlation of BidEL expression with clinicopathological features of patients with adenomatous polyp

The relationships between BidEL expression and clinicopathological variables for an adenomatous polyp in 22 patients were considered. There were no statistically significant associations between BidEL expression and location of polyps (ascending colon, transverse colon, descending colon, sigmoid colon and rectum), although the expression of BidEL in the transverse colon was higher than other polyp’s location (Fig. 2B).

The expression of BidEL isoform in the male group was higher than in the female group but statistically not significant at *P* = 0.526 (Fig. 2C). Also, no correlation was observed between BidEL expression and age (≤ 50-year and > 50-year groups) (*P* = 0.526) (Fig. 2D).

**Fig. 3:**
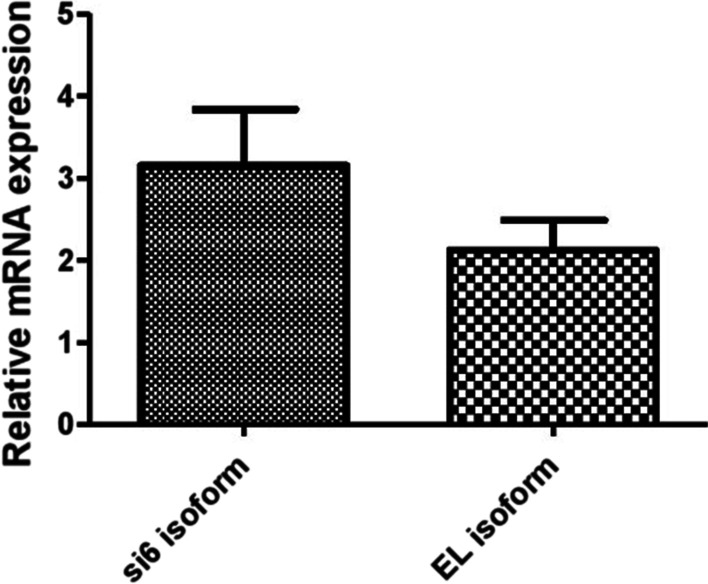
The expression level of BidSi6 and BidEL isoform in polyp tissues. The expression of the BidSi6 isoform was higher than the BidEL isoform in polyps but this difference is not significant (*P* = 0.146)

The expression of Bidsi6 isoform and BidEL isoform were compared in 22 polyps of patients. The expression of the Bidsi6 isoform was higher than the BidEL isoform but statistically not significant at *P* = 0.146 (Fig. 3).

### Diagnostic potential of BidSi6 and BidEL isoform

**Fig. 4:**
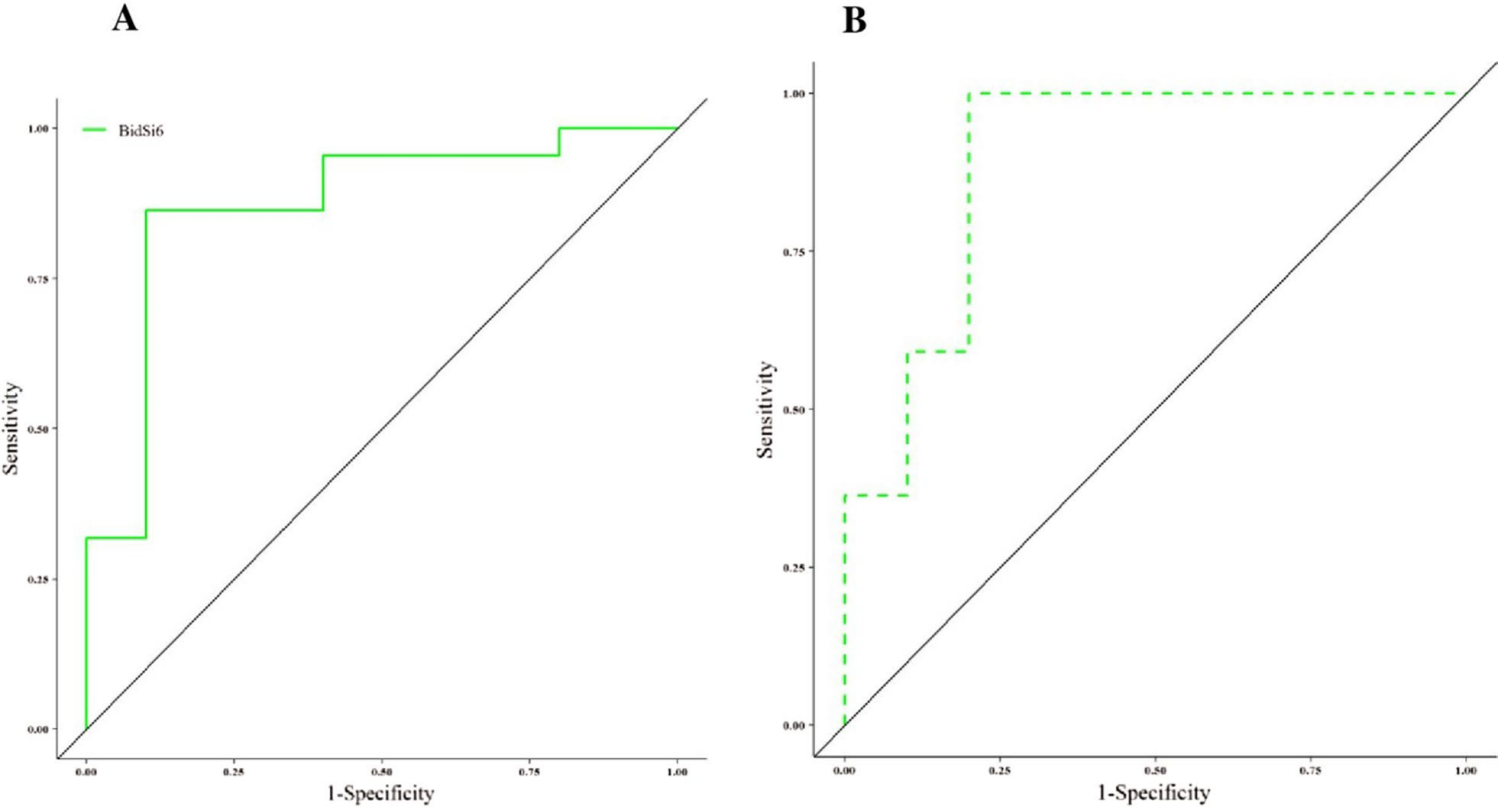
ROC curve analysis of BidSi6 and BidEL isoforms, based on the area under the curve (AUC) in adenomatous polyps to examine the validity of BidSi6 gene expression in discriminating polyps and non-polyps of the colon tissues.** A**. ROC curve analysis of BidSi6 isoforms expression (AUC: 0.8727, AUC CI 0.7255–1, *p*-value: 0.0280).** B**. ROC curve analysis of BidEl isoforms expression (AUC: 0.8955, AUC CI 0.7499–1, *p*-value: 0.0139)

ROC curve analysis was performed to identify the diagnostic value of BidSi6 and BidEL isoform patients with adenomatous polyps (Fig. 4). The AUC value of the ROC curve of the BidSi6 isoform was 0.8727 and 95% confidence interval (CI) was 0.7255–1. The AUC value of the ROC curve of the BidEL isoform was 0.8955 and the 95% confidence interval (CI) was 0.7499–1. These results suggested the potential diagnostic roles of these two isoforms in patients with adenomatous polyps.

### Molecular identification and sequence analysis of the BidSi6 isoform in adenomatous polyp

**Fig. 5:**
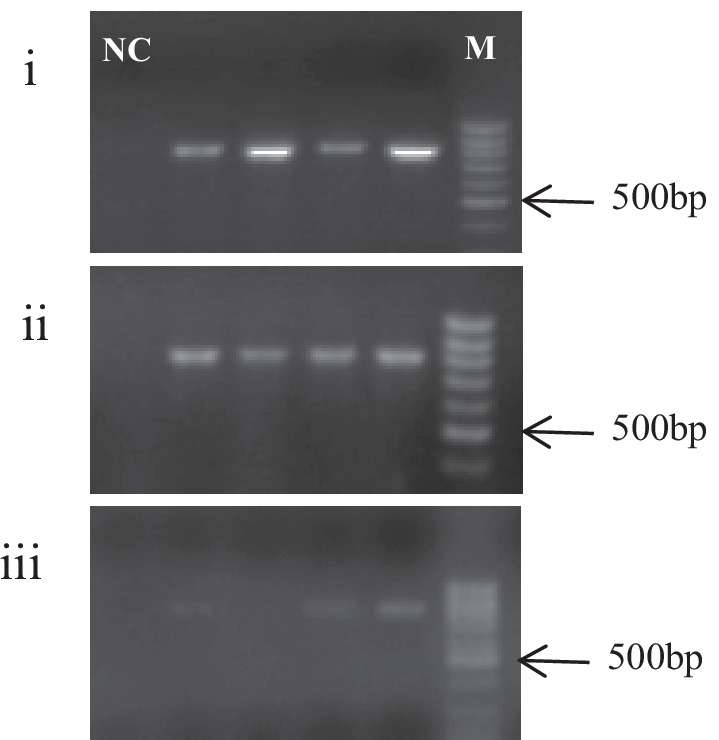
Electrophoresis analysis of PCR products amplified by BidSi6 primers in adenomatous polyp tissues (**i**), adjust non-polyp tissues from the same patients (**ii**) and healthy colorectal tissue (**iii**). The size of the fragment is 777 bp in all individuals. NC: negative control, M: molecular weight marker (100 bp)

**Fig. 6:**
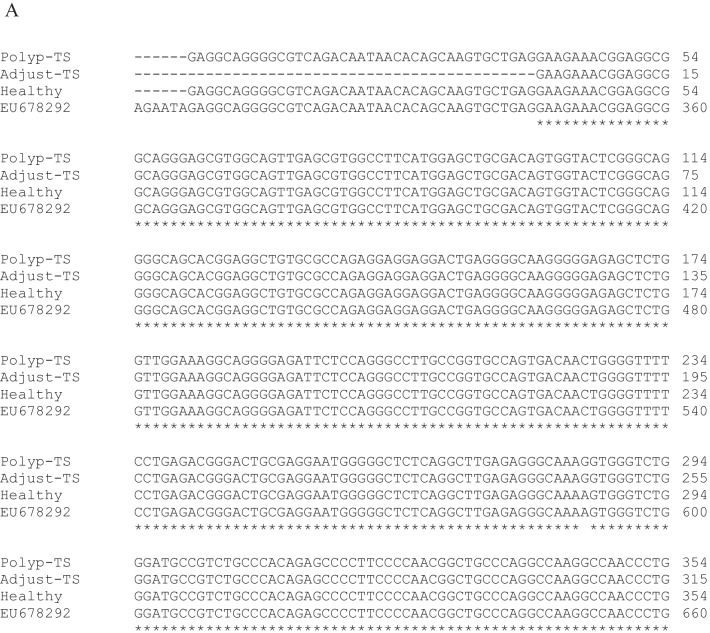

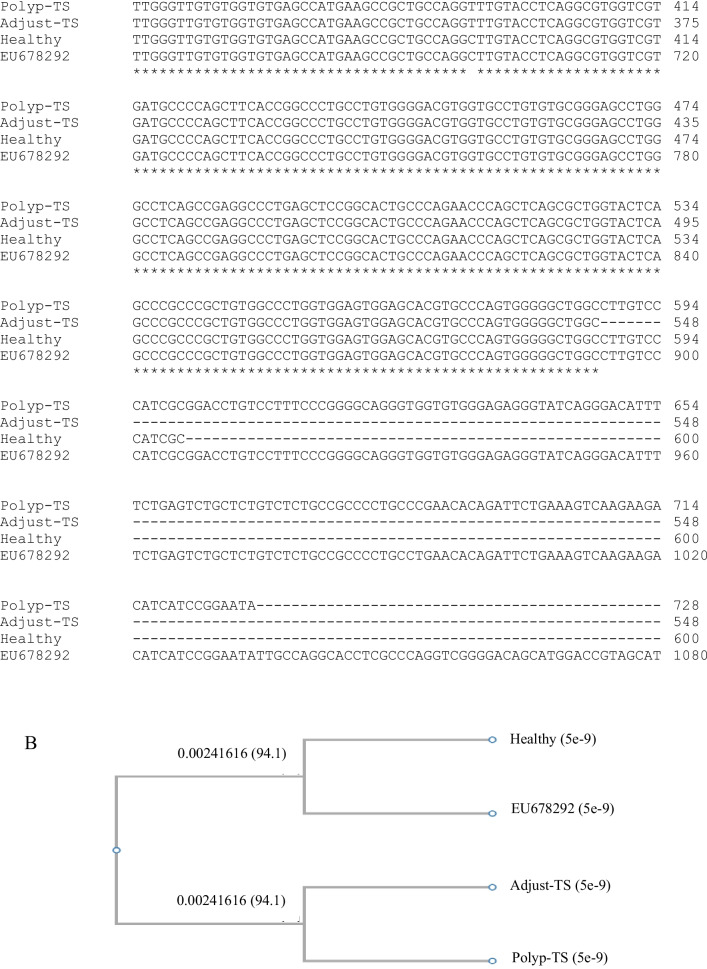
**A** CLUSTAL O (1.2.4) multiple sequence alignment based on the BidSi6 isoform sequence of human adenomatous polyp tissue, adjust non-polyp tissue from the same patient, and healthy colorectal tissue. **B** Phylogenetic tree constructed based on the BidSi6 isoform sequence in human adenomatous polyp tissue, adjust non-polyp tissue from the same patient, healthy colorectal tissue, and reference sequence (EU678292). TS is the code of a sample

Conventional PCR was carried out with BidSi6 primers on adenomatous polyp tissues and adjust non-polyp tissues from the same patients and healthy colorectal tissue. Samples were shown to have PCR amplified fragments with around 777 bp for BidSi6 (Fig. 5). Three PCR positive results of adenomatous polyp tissue, adjust non-polyp tissue from the same patient and healthy colorectal tissue in electrophoresis were selected for sequencing. Sequencing was performed with forward and reverse primers of BidSi6. The sequences were analyzed using the ChromasPro (version 2.6.5) software. All the nucleotide sequences were aligned with each other. According to the identity percentage and query coverage parameter, the reference sequences were downloaded from the GenBank database using the Basic Local Alignment Search Tool (BLAST) (accession number of the reference sequence is EU678292) and Claustal omega (https://www.ebi.ac.uk/Tools/msa/clustalo/), and other programs available in NCBI site (National Center for Biotechnology Information) to determine the isoforms of BidSi6 (Fig. 6A). Phylogenetic tree construction revealed that all the samples stand in a single clade (Fig. 6B).

### Comparison of BidSi6 sequence in adenomatous polyp tissue with BidSi6 reference sequence (EU678292)

**Fig. 7:**
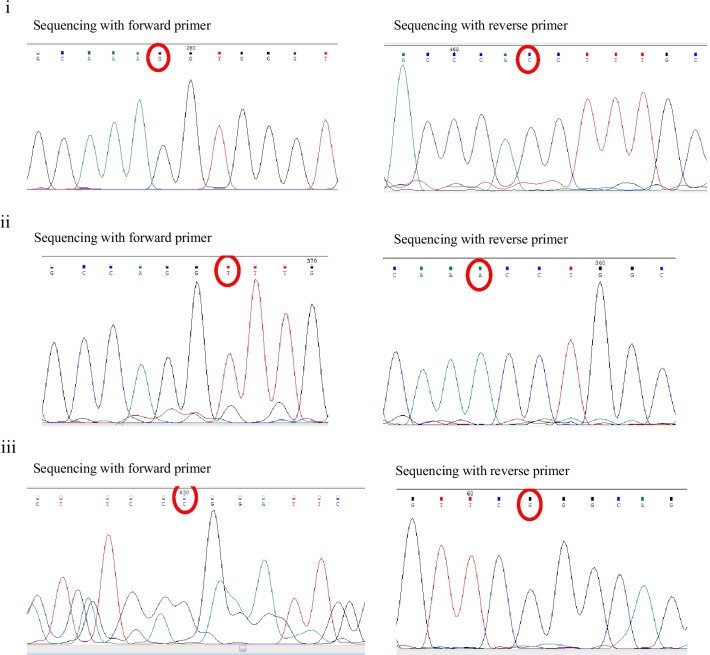
Sequencing of the BidSi6 isoform in human adenomatous polyp tissue with both forward and reverse BidSi6 primers and compared with the reference sequence (EU678292). Three-point mutations were observed in polyp tissue. (**i**) position 591 (A to G), (**ii**) position 699 (C to T), and (**iii**) position 993 (T to C)

The comparison of BidSi6 sequences between human adenomatous polyp tissue and reference sequences (EU678292) using the Basic Local Alignment Search Tool (BLAST) reveals 99% Identities. We found three point mutations in BidSi6 sequences in polyp tissue compared with the reference sequence (EU678292). There are in positions 591 (A to G), 699 (C to T) and 993 (T to C) (Fig. 7), which did not change the amino acid sequence.

Nucleotide and amino acid sequences related to BidSi6 isoform in adenomatous polyp tissue were submitted to GenBank and could be accessed through the following accession numbers: MG957990 and AVP50013 respectively in the European Molecular Biology Laboratory and DNA Data Bank of Japan.

### Comparison of BidSi6 sequence in adjust non-polyp tissue with BidSi6 reference sequence (EU678292)

**Fig. 8:**
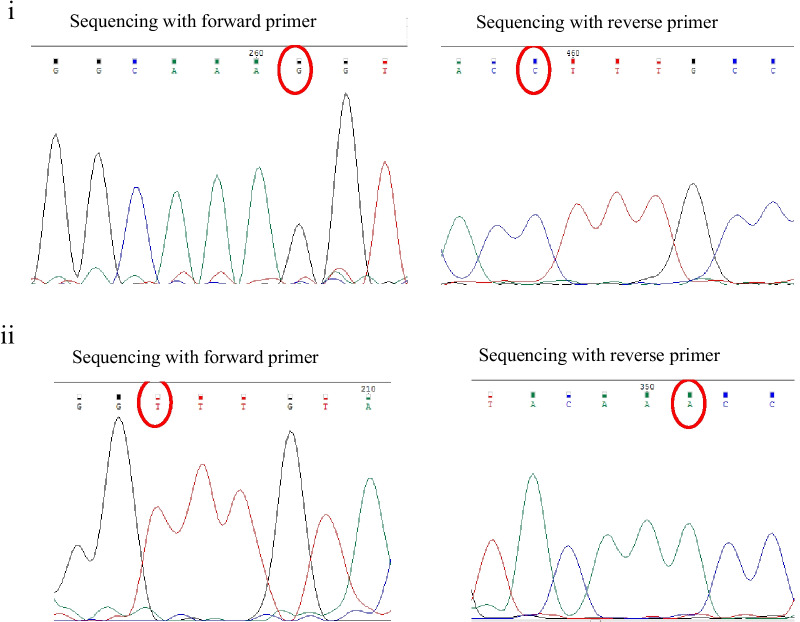
Sequencing of the BidSi6 isoform in the adjust non-polyps tissue with both forward and reverse BidSi6 primers and compared with the reference sequence (EU678292). Two-point mutations were observed in the adjust non-polyps tissue. (**i**) Position 591 (A to G), (**ii**) and position 699 (C to T)

The comparisons of BidSi6 sequences between adjust non-polyp tissue from the same patient mentioned above and reference sequences (EU678292) using the BLAST tool reveal 99% Identities. The alignment of the two sequences reveals two point mutations in BidSi6 sequences in adjust tissue as compared with the reference sequence (EU678292). There are in the same position 591 (A to G) and position 699 (C to T) (Fig. 8) like adenomatous polyp tissue. In this study, the size of the sequenced fragment of BidSi6 isoform in adenomatous polyp tissue and adjust non-polyp tissue from the same patient were 728 bp and 548 bp respectively. Thus, the third point mutation (position 993) in the adjust tissue was not reported. Nucleotide and amino acid sequences related to BidSi6 isoform in adjust non-polyp tissue were submitted to GenBank with the accession numbers MG957991 and AVP50014 respectively in the European Molecular Biology Laboratory and DNA Data Bank of Japan.

### Comparison of BidSi6 sequence in healthy colorectal tissue with BidSi6 reference sequence (EU678292)

The comparisons of BidSi6 sequences between healthy colorectal tissue and reference sequences (EU678292) using the BLAST tool reveals 100% Identities, and no mutations were observed in the BidSi6 sequence of healthy colorectal tissue. Nucleotide and amino acid sequences related to BidSi6 isoform in healthy colorectal tissue were submitted to GenBank, which can be accessed through the following accession numbers: MH121045 and AWQ38328 respectively in the European Molecular Biology Laboratory and DNA Data Bank of Japan.

## Discussion

In the present study, we considered the expression of two isoforms of the Bid gene in adenomatous polyps and adjust non-polyp tissues from the same patient with healthy colorectal tissue. In the second part of our study, we performed molecular identification and sequence analysis of the BidSi6 isoform in the adenomatous polyp, adjust non-polyp tissue and healthy colorectal tissue. More than 95% of colorectal cancers are assumed to have a premalignant adenomatous polyp phase. Approximately there is a 95% increase in colorectal cancers within adenomatous polyps. These lesions with dysplastic epithelium have a great potential for malignancy [16]. During tumorigenesis, cells produce aberrant proteins with inserted, altered or missing functional domains [17]. There is accumulating evidence that aberrant alternative splicing leads to the development and progression of malignancy [18]. Also, we considered the correlation of BidSi6 and BidEL expression with clinicopathological features and its potential as diagnostic and prognostic markers was examined. Aberrant RNA splicing is more effective in drug therapy resistance than previously thought [19]. However, novel classes of biomarkers with high specificity, sensitivity, and efficiency are required for the prognosis and diagnosis of adenomatous polyps.

The bid has a unique role in connecting the activation of the extrinsic apoptosis pathway to the activation of the intrinsic pathway. In addition, Bid has an important role in regulating cell cycle arrest [7, 20].

Alternative splicing introduces another way of control to the gene expression pathways because it produces protein isoforms with approximately different functions [21, 22]. Alternative splicing Bid gene, effects on the endogenous protein level and display segregated functions and cellular localization [9]. Functional Consequences of Alternative Splicing is altered apoptotic potential. Each Bid isoform has behaved differently to change the activity of Bid following cleavage and activation [23–27]. BidEL induces apoptosis, whereas BidS inhibits the apoptotic effects of tBid and abrogates apoptosis mediated by Fas [7, 28]. The features of the BidSi6 isoforms are similar to that of BidS isoforms with an inhibitory BH3B domain that could suppress apoptosis. We found significantly high expression of BidSi6 and BidEL isoforms at mRNA levels in adenomatous polyps and adjust non-polyp tissues from the same patient compared with healthy group, but there was no statistically significant difference between their expression in adenomatous polyps and adjust non-polyp tissues.

Renshaw et al. showed that the expression of BidEL and BidS mRNA level increased during myeloid differentiation. This indicates that BidEL and BidS act differently in the adjustment of the dead cells in developing myeloid cells [9]. Several investigators have suggested that the tBid-N sequence, when expressed separately, can inhibit the pro-apoptotic activity of the tBid-C fragment [9, 29]. Collectively, these data offer a crucial inhibitory effect of the N-terminus of Bid upon the C-terminal pro-apoptotic function during Bid cleavage. Elevated expression of Bid has been reported in some tumors, such as colon carcinomas, gliomas and prostate cancers. Higher levels of Bid were also found in lymphomas with more advanced histology and advanced prostate cancers indicating higher expression compared to earlier stage tumors [6]. Higher levels of Bid may indicate other compensatory defects in apoptosis pathways that help tumor cells to tolerate elevated levels of this protein [28].

Also in the present study, we found that BidSi6 and BidEL isoforms have a relatively high diagnostic efficiency with an AUC of 0.8727 and AUC of 0.8955 respectively for adenomatous polyps. However, BidSi6 and BidEL isoforms were identified as a marker for screening, and they showed high specificity and sensitivity for diagnosis. Moreover, our results demonstrate that the expression of Bidsi6 isoform was higher than BidEL isoform in polyp tissues but statistically not significant. Based on our results, we propose that the upregulation of BidSi6 in patients with Adenoma polyps eliminated the pro-apoptotic effects of tBid and inhibits apoptosis mediated by Fas, which can lead to dysplasia. The presence of pro-apoptotic Bid can also determine tumor cell susceptibility to extrinsic apoptotic stimuli [30] suggesting that both quantity and ratio of Bid splice variants can strongly influence development and chemoresistance in tumor cells [31].

Our findings revealed the diagnostic value of BidSi6 and BidEL isoforms. We studied the relationship between the expression of BidSi6 and BidEL isoforms and clinicopathological factors. However, no significant association was observed between BidSi6 and BidEL isoforms expression and the location of the adenomatous polyp (ascending colon, transverse colon, descending colon, sigmoid colon and rectum), age, and sex (male and female). Earlier studies showed the morphological and clinical features of colon and rectum cancers [32–34]. Colon and rectum cancers are different in many respects such as the rate and pattern of the mutations and inheritance [35]. On the contrary, this study showed that the polyps of the colon expressed a similar mRNA mold to those observed in the rectum.

In the second part of this study, we performed sequencing of BidSi6 in adenomatous polyp tissue, adjust non-polyp tissue from the same patients, and healthy colorectal tissue. It was revealed that there was 99% homology between BidSi6 sequences in a human adenomatous polyp tissue, adjust and the reference sequence (EU678292). The phylogenetic tree was constructed based on the sequence of idSi6 isoform readily separated adenomatous polyp tissue and adjust non-polyp tissue from healthy colorectal tissue and the reference sequence (EU678292).

## Conclusion

Our study found significant upregulation of BidSi6 and BidEL isoforms in adenomatous polyp and its adjacent non-polyp tissues compared to that in the normal colon tissue. Alternative splicing events were shown to provide possible therapeutic targets for colorectal polyps. Our findings suggest that these two isoforms of Bid may be a novel diagnostic biomarker for the identification of the adenomatous polyp. However, several studies are needed to know the prognostic value of BidSi6 and BidEL isoforms.

## Supplementary Information

**Additional file 1.** Location of the BidSi6 and BidEL amplified product in Bid isoform EL and Si6 mRNA sequences. In this study primers of BidSi6 and BidEL were designed according to GenBank reference sequences of these genes in NCBI with accession numbers EU678292 and AF250233 respectively. Forward and reverse primers were shown in bold and underlined. The size of the amplified product by BidSi6 and BidEL primers are 157bp and 138bp respectively.

## Data Availability

The accession numbers of nucleotide and amino acid sequences related to BidSi6 isoform in adenomatous polyp tissue that we submitted to GenBank are MG957990 (https://www.ncbi.nlm.nih.gov/nuccore/MG957990) and AVP50013 (https://www.ncbi.nlm.nih.gov/protein/AVP50013) respectively. Also, the accession numbers nucleotide and amino acid sequences related to BidSi6 isoform in healthy colorectal tissue submitted to GenBank are MH121045 (https://www.ncbi.nlm.nih.gov/nuccore/MH121045) and AWQ38328 (https://www.ncbi.nlm.nih.gov/protein/AWQ38328) respectively.
