# Supplementary material for: BidSi6 and BidEL isoforms as a potential marker for predicting colorectal adenomatous polyps
Source: BMC Med Genomics. 2022 Jun 6;15:129. doi: 10.1186/s12920-022-01282-0 (PMC9172139; doi:10.1186/s12920-022-01282-0)
Supplement: Supplementary file 1 — Additional file 1. Location of the BidSi6 and BidEL amplified product in Bid isoform EL and Si6 mRNA sequences. In this study primers of BidSi6 and BidEL were designed according to GenBank reference sequences of these genes in NCBI with accession numbers EU678292 and AF250233 respectively. Forward and reverse primers were shown in bold and underlined. The size of the amplified product by BidSi6 and BidEL primers are 157bp and 138bp respectively. [file 12920_2022_1282_MOESM1_ESM.docx]

**Additional file 1**

>gb|EU678292.1| Homo sapiens BID isoform Si6 mRNA, complete cds

GGACGCGCCCGCGCCCCCGCGGCTGGAGGGTGGTCGCCACTGGGACACTGTGAACCAGGAGTGAGTCGGA

GCTGCCGCGCTGCCCAGGCCATGGACTGTGAGGTCAACAACGGTTCCAGCCTCAGGGATGAGTGCATCAC

AAACCTACTGGTGTTTGGCTTCCTCCAAAGCTGTTCTGACAACAGCTTCCGCAGAGAGCTGGACGCACTG

GGCCACGAGCTGCCAGTGCTGGCTCCCCAGTGGGAGGGCTACGATGAGCTGCAGACTGATGGCAACCGCA

GCAGCCACTCCCGCTTGGGAAG**AATAGAGGCAGGGGCGTC**AGACAATAACACAGCAAGTGCTGAGGAAGA

*AACG*GAGGCGGCAGGGAGCGTGGCAGTTGAGCGTGGCCTTCATGGAGCTGCGACAGTGGTACTCGGGCAG

GGGCAGCACGGAGGCTGTGC**GCCAGAGGAGGAGGACTGA**GGGGCAAGGGGGAGAGCTCTGGTTGGAAAGG

CAGGGGAGATTCTCCAGGGCCTTGCCGGTGCCAGTGACAACTGGGGTTTTCCTGAGACGGGACTGCGAGG

AATGGGGGCTCTCAGGCTTGAGAGGGCAAAAGTGGGTCTGGGATGCCGTCTGCCCACAGAGCCCCTTCCC

CAACGGCTGCCCAGGCCAAGGCCAACCCTGTTGGGTTGTGTGGTGTGAGCCATGAAGCCGCTGCCAGGCT

TGTACCTCAGGCGTGGTCGTGATGCCCCAGCTTCACCGGCCCTGCCTGTGGGGACGTGGTGCCTGTGTGC

GGGAGCCTGGGCCTCAGCCGAGGCCCTGAGCTCCGGCACTGCCCAGAACCCAGCTCAGCGCTGGTACTCA

GCCCGCCCGCTGTGGCCCTGGTGGAGTGGAGCACGTGCCCAGTGGGGGCTGGCCTTGTCCCATCGCGGAC

CTGTCCTTTCCCGGGGCAGGGTGGTGTGGGAGAGGGTATCAGGGACATTTTCTGAGTCTGCTCTGTCTCT

GCCGCCCCTGCCTGAACACAGATTCTGAAAGTCAAGAAGACATCATCCGGAATATTGCCAGGCACCTCGC

CCAGGTCGGGGACAGCATGGACCGTAGCATCCCTCCGGGCCTGGTGAACGGCCTGGCCCTGCAGCTCAGG

AACACCAGCCGGTCGGAGGAGGACCGGAACAGGGACCTGGCCACTGCCCTGGAGCAGCTGCTGCAGGCCT

ACCCTAGAGACATGGAGAAGGAGAAGACCATGCTGGTGCTGGCCCTGCTGCTGGCCAAGAAGGTGGCCAG

TCACACGCCGTCCTTGCTCCGTGATGTCTTTCACACAACAGTGAATTTTATTAACCAGAACCTACGCACC

TACGTGAGGAGCTTAGCCAGAAATGGGATGGACTGAACGGACAGTTCCAGAAGTGTGACTGGCT

>gb|AF250233.1| Homo sapiens Bid isoform EL mRNA, complete cds

ACCCTTGCCGGCGGATCGGAATCCCCGCCCACACCGTGGTCTTTCCAGCACCGCAGACACCTGCCGGCTC

CTCCCGAGCGGAGCTCAGGGCTGACAAGGCGCGGTCAGAGCGGCCGCTTACTGGGGCTCGCCCGCTCCTT

AGAGCACTGGCAATGATGTGCGGATCCTCGCTGCTGCTGCTGGGAAACTGTTGAGTGGCTGAATGACCCC

AGGGGACCCTGGGAGAGCTCTGAAGCCCTCAGCCACC**AAGTGGCTGGGCTGGCAAG**GGTTCATTCATTCA

TTCAACAAATACGAATGTGCAGCGGTGCTGGGGTCATGATGGCTCGGTGGGCAGCGAGGGGCCGGGCCGG

CTGGAGGAGCACAGTG**CGGATTCTGTCGCCACTGG**GACACTGTGAACCAGGAGTGAGTCGGAGCTGCCGC

GCTGCCCAGGCCATGGACTGTGAGGTCAACAACGGTTCCAGCCTCAGGGATGAGTGCATCACAAACCTAC

TGGTGTTTGGCTTCCTCCAAAGCTGTTCTGACAACAGCTTCCGCAGAGAGCTGGACGCACTGGGCCACGA

GCTGCCAGTGCTGGCTCCCCAGTGGGAGGGCTACGATGAGCTGCAGACTGATGGCAACCGCAGCAGCCAC

TCCCGCTTGGGAAGAATAGAGGCAGATTCTGAAAGTCAAGAAGACATCATCCGGAATATTGCCAGGCACC

TCGCCCAGGTCGGGGACAGCATGGACCGTAGCATCCCTCCGGGCCTGGTGAACGGCCTGGCCCTGCAGCT

CAGGAACACCAGCCGGTCGGAGGAGGACCGGAACAGGGACCTGGCCACTGCCCTGGAGCAGCTGCTGCAG

GCCTACCCTAGAGACATGGAGAAGGAGAAGACCATGCTGGTGCTGGCCCTGCTGCTGGCCAAGAAGGTGG

CCAGTCACACGCCGTCCTTGCTCCGTGATGTCTTTCACACAACAGTGAATTTTATTAACCAGAACCTACG

CACCTACGTGAGGAGCTTAGCCAGAAATGGGATGGACTGAACGGACAGTTCCAGAAGTGTGACTGGCTAA

AGCTTGATGTGGTCACAGCTGTATAGCTGCTTCCAGTGTAGACGGAGCCCT

Location of the BidSi6 and BidEL amplified product in Bid isoform EL and Si6 mRNA sequences. In this study primers of BidSi6 and BidEL were designed according to GenBank reference sequences of these genes in NCBI with accession numbers EU678292 and AF250233 respectively. Forward and reverse primers were shown in bold and underlined. The size of the amplified product by BidSi6 and BidEL primers are 157bp and 138bp respectively.
